# Tagging single nucleotide polymorphisms in the *PPAR-γ* and *RXR-α* gene and type 2 diabetes risk: a case-control study of a Chinese Han population^☆^

**DOI:** 10.1016/S1674-8301(11)60004-3

**Published:** 2011-01

**Authors:** Juan Du, Hui Shi, Ying Lu, Wencong Du, Yuanyuan Cao, Qian Li, Jianhua Ma, Xinhua Ye, Jinluo Cheng, Xiaofang Yu, Yanqin Gao, Ling Zhou

**Affiliations:** a Department of Epidemiology and Biostatistics, School of Public Health, Nanjing Medical University, Nanjing, Jiangsu 210029, China;; b Department of Endocrinology, the Affiliated Nanjing First Hospital of Nanjing Medical University, Nanjing, Jiangsu 210006, China;; c Department of Endocrinology, the Affiliated Changzhou Second Hospital of Nanjing Medical University, Changzhou, Jiangsu 213003, China;; d Department of Endocrinology, the Affiliated Yizheng Third Hospital of Nanjing Medical University, Yizheng, Jiangsu, 211900 China.

**Keywords:** peroxisome proliferators-activated receptor-γ, retinoid X receptor-α, type 2 diabetes mellitus, single nucleotide polymorphism, serum adiponectin

## Abstract

Peroxisome proliferator-activated receptor (PPAR-γ),which is mainly involved in adipocyte differentiation, has been suggested to play an important role in the pathogenesis of insulin resistance and atherosclerosis. We investigated the frequencies of two common tagging polymorphisms of the *PPAR*-*γ* gene and two of *PPAR*-*α* with minor allele frequency (MAF) ≥0.05 in the Chinese Han population and analyzed the correlation between the different genotypes and the risk of type 2 diabetes mellitus (T2DM). *Taq*Man® assay was performed to test the genotypes in T2DM patients (*n* = 1,105) and normal controls (*n* = 1,107). Serum adiponectin concentration was measured by ELISA kit. The variant genotypes rs17817276GG, rs3856806CT and rs3856806CT/TT of *PPAR*-*γ* were associated with T2DM, *P* = 0.023,0.037 and 0.018, respectively. Furthermore, the prevalence of haplotype GT in *PPAR*-*γ* was less frequent in the case subjects (0.3%) than in the controls (1.9%) [*P* < 0.001,*OR*(95%*CI*)=0.13 (0.06-0.31)]. Patients with genotype TT of rs3856806 had a higher serum level of adiponectin than those with the genotype CC and CT (*P* = 0.031 and 0.038, respectively). There was no statistically significant difference between patients and controls in genotype distribution of rs6537944 and rs1045570 of the *RXR*-*α* gene. The present study suggests that the variant genotypes in the *PPAR*-*γ* gene could decrease the risk for the development of T2DM in the Chinese Han population.

## INTRODUCTION

Type 2 diabetes mellitus (T2DM), which is a metabolic disorder characterized by insulin resistance and pancreatic β-cell dysfunction, is a leading cause of morbidity and mortality worldwide. Recently, adiponectin has also been reported as a new risk factor in diabetes development. The adipose tissue exclusively secretes adiponectin, a 244-amino-acid protein that regulates the metabolism of lipids and glucose and circulates quite abundantly in plasma[1],[2]. Adiponectin is an adipocytokine we identified from human fat cDNA[3]. Mouse homolog for adiponectin, Acrp30 and AdipoQ, was cloned by Scherer *et al*.[4] and Hu *et al*.[5], independently. Adiponectin exists abundantly in human blood (5-20 µg/mL), and its plasma concentration decreases with fat accumulation in the body[6].Plasma adiponectin concentrations are lower in patients with diabetes[7] and ischemic heart disease[8]. Significantly, the genetic mutation of the adiponectin gene, which causes low plasma adiponectin levels, is associated with the metabolic syndrome, including insulin-resistant diabetes and atherosclerotic disease[9]. The current views indicate that thiazolidinediones, as peroxisome proliferators-activated receptor-γ (PPAR-γ) agonists, have been shown to increase plasma adiponectin levels by transcriptional induction in adipose tissues. *PPAR*-*γ*/retinoid X receptor (*RXR*) heterodimer directly binds to the functional PPAR-responsive element (*PPRE*) and increased the promoter activity in cells. Adipocyte expression of the adiponectin gene is maintained and induced by binding endogenous or exogenous *PPAR*-*γ*/*RXR* heterodimer to the *PPRE* (-273/-285) in adiponectin promoter[10],[11]. *PPAR*-*γ*, a member of the nuclear hormone receptor subfamily of transcription factors, is involved in the expression of target genes implicated in adipocyte differentiation and glucose homeostasis[12],[13]. The genes encoding PPAR-γ and RXR-α that are located in the adiponectin pathway are therefore candidates for T2DM and obesity. In this study, we used the tagging single nucleotide polymorphism (SNP) strategy to examine potential associations among genetic variants in the *PPAR*-*γ* and *RXR*-*α* gene with diabetes risk in a Chinese population.

SNPs in the *PPAR*-*γ* gene have been reported to be associated with the risk of diabetes[12],[14],[15]. But at present, there are only a few such studies on SNPs in the *RXR*-*α* gene. Because only one SNP might not represent genetic variation in genes, we tested the hypothesis that multiple potentially tagging SNPs of the *PPAR*-*γ* and *RXR*-*α* gene were associated individually or jointly with risk of T2DM in a case-control study with a moderate sample size (1,105 cases and 1,107 controls) in a Chinese Han population.

## MATERIALS AND METHODS

### Subjects

Our study included 1,105 T2DM patients and 1,107 T2DM-free controls. All subjects were genetically unrelated ethic Han Chinese, and each gave informed consent. The cases included all eligible patients newly diagnosed with T2DM according to the diagnostic criteria of the World Health Organization (WHO) for diabetes, who were consecutively recruited between Mar 2008 and Aug 2010 at the diabetes outpatient clinic from three affiliated hospitals of Nanjing Medical University (The Affiliated Changzhou Second Hospital, the Affiliated Yizheng Third Hospital and the Affiliated First Hospital). Patients were recruited reqardless of age and sex (536 males and 569 females; aged 57.07±11.11 years). A diagnosis of T2DM required either fasting plasma glucose (FPG)≥ 7.0 mmol/L (126 mg/dL) or 2h glucose≥11.1 mmol/L (200 mg/dL) after an oral glucose tolerance test[16]. All the patients were tested by glutamic acid decarboxylase autoantibodies (GAD) and islet-cell antibodies (ICA512) (RSI Company, UK) to exclude patients with type 1 diabetes. T2DM-free controls were randomly selected from outpatient clinics within the same geographical area and during the period when the cases were recruited. Controls (516 males and 591 females, mean age 57.02±11.40 years) were enrolled following routine annual health examinations, and were determined to be non-diabetic by an oral glucose tolerance test (75 g of glucose), performed according to the WHO criteria. These control subjects had no history of T2DM and were frequency-matched to the cases on the base of age (±5 years) and sex.

### Demographic data collection

Each participant was scheduled for an interview and a structured questionnaire was administered by interviewers to collect information on demographic data and environmental exposure history including tobacco smoking and drinking. After the interview, an approximate 5 mL venous blood sample was collected from each participant.

### Measurements

Weight, height, waist circumference (WC) and hip circumference (HC) were measured by trained personnel, and the body mass index (BMI) was calculated. Seated blood pressure (BP) was measured on the right arm with the individual in a sitting position after 10 min rest using a standard sphygmomanometer of appropriate cuff size. After an overnight fast, venous blood samples were collected and promptly centrifuged, and the serum was stored at -20°C until an adiponectin assay was performed. All samples were performed in the same assay. Serum adiponectin was measured by ELISA (human adiponectin ELISA kit; RapidBio Company, USA). FPG was measured in the laboratories in the three affiliated hospitals of Nanjing Medical University using the glucose oxidase method. Total cholesterol, high-density lipoprotein (HDL) cholesterol, low-density lipoprotein (LDL) cholesterol and triglycerides were determined in the three affiliated hospitals by an enzymatic colorimetric method (Au5400, Olympus, Japan). DNA was extracted from peripheral blood using proteinase K and phenol/chloroform. This study was approved by the Research Ethics Committee of Nanjing Medical University and informed consent was obtained from all study participants.

### Tagging SNP selection and genotyping assays

We used the public HapMap SNP database (http://www.hapmap.org) to identify *PPAR*-*γ* and *RXR*-*α* gene tagging SNPs by using tagger with a greedy algorithm. The tagging SNPs were selected on the basis of their pairwise linkage disequilibrium with the *r*^2^ threshold of 0.8 and minor allele frequency (MAF) ≥0.05 to capture all common SNPs. Finally, we respectively found four SNPs (rs17817276, rs3856806, rs6537944, rs1045570) located in the DNA-binding domain or ligand-binding domain in the cDNA.

Genomic DNA was extracted from peripheral blood samples of all subjects. 5′-Nuclease *Taq*Man® assays were used to genotype the polymorphisms in 384-well plates on ABI PRISM 7900HT Sequence Detection system (Applied Biosystems, Foster City, CA, USA). The primers and probes of *Taq*Man® assays were designed using Primer Express Oligo Design software v2.0 (ABI PRISM) and available upon request as *Taq*Man® Pre-Designed SNP Genotyping Assays. The primer and probe sequences used are shown in ***Table 1***. PCR was performed in a 5 µL reaction mixture containing 5 ng DNA, 2.5 µL 2× *Taq*Man® Universal PCR Master Mix and 0.083 µL 40× Assay Mix. The PCR conditions were 50°C for 2 min, 95°C for 10 min, 95°C for 15 s and then 60°C for 1 min, and forty cycles of real-time PCR were performed. Individual genotype identification was performed by SDS software 2.0 (ABI, USA). Each plate contained 2 samples from the same individual as positive controls and 2 blank samples as negative controls for the genotyping quality confirmation. In addition, 10% of the samples were randomly selected to perform repeated assays; the results were 100% concordant.

**Table 1:** Primer and probe sequences for the amplification of SNPs in the *PPAR*-*γ* and *RXR*-*α* gene

SNP		Primers and probes (5′'-3′)
rsl7817276	Sense	CTCCCTGACAGCAGCTATCC
	Antisense	TTCCCAGGATTATCCTAACAGA
	Probe 1	AAATAGTAATATATGACAACCT
	Probe 2	AATAGTAATACATGACAACC
rs3856806	Sense	TGTTTGCCAAGCTGCTCC
	Antisense	TTGGCAGTGGCTCAGGAC
	Probe 1	CTGCACGTGTTCC
	Probe 2	CTGCACATGTTCC
rs6537944	Sense	CGTGAATGCTGCTCTCTCTGT
	Antisense	AACTGGATATGGGCAGCACT
	Probe 1	CGTTCCGTCAGGCA
	Probe 2	CGTTCCATCAGGCA
Rs1045570	Sense	AGCCTTGCTCTGTTGTGTCC
	Antisense	ACTTCTCCCTTTGCGTGTTC
	Probe 1	CACCTGCGGCCAC
	Probe 2	CACCTGAGGCCAC

### Statistical analysis

The distribution of the general characteristic and genotype frequencies between the T2DM cases and T2DM-free controls was compared by using two-tailed χ^2^-test or Student's *t*-tests. Among controls, genotype frequencies for each SNP were tested for Hardy-Weinberg equilibrium by a Linear Discriminant Analysis (LDA)[17]. The associations among *PPAR-γ* and *RXR-α* gene genotypes and T2DM were estimated by computing the odds ratios (ORs) and 95% confidence intervals (95%CIs) from both univariate and multivariate logistic regression analysis with adjustment for age, sex and BMI. Haplotypes were predicted using the PHASE (v 2.1) Bayesian algorithm[18]. A two-tailed *P* value less than 0.05 was considered statistically significant. All the statistical analyses were performed by SPSS software (Version 13.0, SPSS INC, USA). 

## RESULTS

### Subjects' characteristics

The distribution of selected characteristics between T2DM patients and controls are summarized in ***Table 2***. Among the selected characteristics, significant differences existed between cases and controls in blood pressure, HDL-C, total cholesterol, triglycerides, FPG and adiponectin level.

**Table 2:** Demographic and clinical characteristics of the study population

Variables	Cases (*n* = 1,105)	Controls (*n* = 1,107)	*P*
Sex (male/female)	536/569	516/591	0.372
Age (years)	57.07 ±11.11	57.02 ± 11.40	0.380
Systolic pressure (mmHg)	137.99 ±20.56	127.93 ± 18.13	< 0.001
Diastolic pressure (mmHg)	85.02 ±11.80	79.35 ± 10.42	0.002
BMI (kg/m^2^)	24.88 ± 3.55	24.15 ± 3.27	0.069
HDL-C (mmol/L)	1.14 ±0.46	1.42 ± 0.37	0.017
LDL-C (mmol/L)	2.82 ± 0.96	2.57 ± 0.89	0.062
Total cholesterol (mmol/L)	5.16 ±1.37	5.04 ± 0.95	< 0.001
Triglyceride (mmol/L)	2.62 ± 2.75	1.60 ± 1.04	< 0.001
Fasting plasma glucose (mmol/L)	10.91 ± 3.94	5.08 ± 0.53	< 0.001
Adiponectin (mg/L)	6.23 ± 1.74	7.14 ± 2.62	< 0.001

BMI: body mass index; HDL: high density lipoprotein; LDL: low density lipoprotein

(mean±SD)

### Subjects' genotypes

The genotype distribution for all the SNPs did not show any deviation from the Hardy-Weinberg equilibrium in the control groups (*P* = 0.118, 0.343, 0.108 and 0.458). Among the 2,212 subjects, the successfully genotyped rates for the 4 SNPs were all more than 95%. The allele frequencies of *rs3856806* and *rs17817276* in the *PPAR*-*γ* gene were statistically different between cases and controls (*P* = 0.037 and 0.014, respectively, ***Table 3***). There were no significant differences in terms of *rs6537944* and *rs1045570* of the *RXR*-*α* gene between the two groups. Logistic regression analysis revealed that, compared with the *rs3856806CC* genotype, subjects carrying the heterozygous *rs3856806CT* genotype had a significantly decreased risk of T2DM [adjusted *P* = 0.031, *OR* (95% *CI*) = 0.82(0.68 - 0.98)] and the combined rs3856806CT/TT variant genotypes were associated with a significantly decreased risk of diabetes [adjusted *P* = 0.016, *OR* (95% *CI*) = 0.81(0.68-0.96)]. Similarly, compared with the *rs17817276AA* wild-type genotype, genotype *rs17817276GG* was associated with a decreased risk of T2DM [adjusted *P* = 0.010, *OR* (95%*CI*) = 0.47(0.27-0.83)]. However, none of the two polymorphisms in the *RXR*-*α* gene, *rs6537944* or *rs1045570*, showed significant associations with T2DM in this study population.

**Table 3:** The distribution of genotypes in the sudy prpulation

Genotype	Cases *n* (%)	Controls *n* (%)	Crude OR *OR*(*95%CI*)	*P*	Adjusted *OR*(*95% CI*)	Adjusted P*
rs17817276	1100	1106				
AA	804 (73.1)	775 (70.1)	1.00	N/A	1.00	N/A
AG	275 (25.0)	293 (26.5)	0.91(0.75-1.10)	0.306	0.92(0.75-1.12)	0.387
GG	21 (1.9)	38 (3.4)	0.53(0.31-0.92)	0.023	0.47(0.27-0.83)	0.010
AG/GG	296 (26.9)	331 (29.9)	0.99(0.82-1.19)	0.871	0.98(0.81-1.19)	0.842
G allele	317 (14.4)	369 (16.7)	0.84(0.71-0.99)	0.037		
A allele	1,883 (85.6)	1,843 (83.3)				
rs3856806	1,105	1,107				
CC	666 (60.3)	612 (55.3)	1.00		1.00	
CT	373 (33.8)	414 (37.4)	0.83(0.63-0.99)	0.037	0.82(0.68-0.98)	0.031
TT	66 (6.0)	81 (7.3)	0.75(0.53-1.06)	0.098	0.75(0.53-1.07)	0.114
CT/TT	439 (39.8)	495 (44.7)	0.82(0.69-0.97)	0.018	0.81(0.68-0.96)	0.016
T allele	505 (22.9)	576 (26.0)	0.82(0.73-0.97)	0.014		
C allele	1,705 (77.1)	1,638 (74.0)				
rs6537944	1,089	1,103				
CC	16 (1.5)	11 (1.0)	1.00	N/A	1.00	N/A
C	243 (22.3)	250 (22.7)	1.48(0.68-3.20)	0.324	1.56(0.72-3.39)	0.265
TT	830 (76.2)	842 (76.3)	0.99(0.81-1.21)	0.891	1.05(0.85-1.29)	0.644
CT/TT	259 (23.8)	261 (23.7)	0.99(0.82-1.21)	0.947	0.93(0.76-1.14)	0.499
T allele	1,903 (87.4)	1,934 (87.7)	0.97(0.81-1.16)	0.767		
C allele	275 (12.6)	272 (12.3)				
rs1045570	1,088	1,106				
GG	712 (65.4)	718 (64.9)	1.00		1.00	
GT	332 (30.5)	351 (31.7)	0.95(0.80-1.15)	0.612	0.99(0.82-1.20)	0.942
TT	44 (4.0)	37 (3.3)	1.20(0.77-1.88)	0.428	1.12(0.70-1.77)	0.645
GT/TT	376 (34.5)	388 (35.0)	0.98(0.82-1.17)	0.797	1.01(0.84-1.16)	0.954
T allele	420 (19.3)	425 (19.2)	1.01(0.87-1.17)	0.941		
G allele	1,756 (80.7)	1,787 (80.8)				

*Adjusted for age, sex and body mass index (BMI). OR: odds ratio; CI: contidence interval.

### Haplotype analysis

PHASE (v 2.1) was used to determine haplotypes. The total number was 2,206 of *PPAR*-*γ* and 2,181 of *RXR*- *α*, excluding the unsuccessfully genotyped subjects in the SNPs. In the *PPAR*-*γ* gene, compared with the most common haplotype AC, haplotype GT was less common in cases than in the controls (0.3% and 1.9%, respectively). The GT haplotype was associated with a 0.13-fold decreased risk of diabetes [ajusted *P* < 0.001, *OR* (95%*CI*) = 0.13(0.06-0.31)]. However, in the *RXR*-*α* gene, compared with the most common haplotype TG, there were no significant differences with haplotype TT, CG, CT (***Table 4*** and ***Table 5***).

**Table 4:** *ORs* and 95%*CIs* for the association between inferred *PPAR*-*γ* haplotypes and diabetes in the study population

Haplotypes	Case (*n* = 1,100)		Control (*n* = 1,106)		*P*^a^	*p*^b^	Adjusted^c^ *OR*(95% *CI*)
	Chromosome No.	%	Chromosome No.	%			
AC	1,387	63.0	1,309	59.2	—	—	—
AT	496	22.5	534	24.1	0.072	0.076	0.874 (0.754-1.014)
GC	311	14.1	328	14.8	0.207	0.209	0.892 (0.747-1.066)
GT	6	0.3	41	1.9	0.000	0.000	0.130 (0.055-0.308)

a: Loci of single nucleotide polymorphisms (SNPs) are written from 5′ to 3′ and include the following SNPs: rs17817276, rs3856806; b: adjusted *P* value; c: adjusted for age, gender and body mass index (BMI). CI: contidence interval; OR: odds ratio.

**Table 5:** *ORs* and 95% *CIs* for the association between inferred *RXR*-*α* haplotypes and diabetes in the case-control study

Haplotypes	Case *(n* = 1,078)		Control *(n* = 1,103)		*P*^a^	*p*^b^	Adjusted^c^ *OR*(95% *CI*)
	Chromosome No.	%	Chromosome No.	%			
TG	1,481	68.7	1,524	69.1	—	—	—
TT	405	18.8	410	18.6	0.836	0.685	1.03 (0.88-1.21)
CG	260	12.0	261	11.8	0.794	0.392	1.09 (0.90-1.32)
CT	10	0.5	11	0.5	0.947	0.989	0.99 (0.42-2.36)

a: Loci of single nucleotide polymorphimus (SNPs) are written from 5′ to 3′ and include the following SNPs: rs1045570, rs6537944; b: Adjusted *P* value; c: adjusted for age, gender and body mass index (BMI). CI: contidence interval; OR: odds ratio.

### Adiponectin level of different genotypes of the *PPAR*-*γ* and *RXR*-*α* gene in the study population

As presented in ***Table 6***, patients with genotype TT of rs3856806 had higher levels of serum adiponectin than those with the genotype CC and genotype CT (*P* = 0.031 and 0.038). There was no significant association in the other three SNPs.

**Table 6:** Adiponectin level of different genotypes of rs17817276, rs3856806, rs6537944 and rs1045570 in the study population

SNPs	Cases		Controls	
	No.	mean±SD	No.	mean ± SD
rs17817276	1,100		1,106	
AA	804	6.18 ±1.76	775	7.11 ± 2.64
AG	275	6.30 ±1.72	293	7.17 ± 2.62
GG	21	6.86 ±1.32	38	7.56 ± 2.28
* P*	N/A		N/A	
rs3856806	1,105		1,107	
CC	666	6.20 ±1.80	612	7.15 ± 2.67
CT	373	6.20 ±1.67	414	7.20 ± 2.64
TT	66	6.68 ±1.46	81	6.77 ± 2.09
* P*	0.031^a^,0.038^b^		N/A	
rs6537944	1,089		1,103	
CC	16	6.77 ±1.45	11	6.79 ± 1.53
CT	243	6.18 ±1.83	250	7.10 ± 2.70
TT	830	6.22 ±1.73	842	7.16 ± 2.61
* P*	N/A		N/A	
Rs1045570	1,088		1,106	
GG	712	6.20 ±1.79	718	7.18 ± 2.62
GT	332	6.21 ± 1.63	351	7.05 ± 2.61
TT	44	6.21 ± 1.63	37	7.31 ± 2.75
* P*	N/A		N/A	

a: *vs* CC genotype of adiponectin level from ANOVA in cases; b: *vs* CT genotype of adiponectin level from ANOVA in cases.

### The stratified analysis of the *PPAR*-*γ* gene

The stratified analysis of rs3856806 in the *PPAR*-*γ* gene showed that a more pronounced reduction in T2DM risks was observed in male and younger individuals (≤50-year-old) carrying the *rs3856806TT* genotype [*OR*(95%*CI*)=0.56(0.33-0.93), 0.25(0.13-0.50), respectively]. Similarly, *rs3856806CT* decreased the risk of T2DM in the elderly (>50-year-old) and obese individuals [*OR*(95%*CI*)=0.81(0.65-0.99), 0.49(0.29-0.83), respectively, ***Table 7***]. Through analysis of *rs17817276* in the *PPAR*-*γ* gene, we found that there were more pronounced reductions in T2DM risks in male and old individuals carrying the *rs17817276GG* genotype [*OR*(95%*CI*)=0.36(0.16-0.80), 0.48(0.26-0.91)]. Males carrying *rs17817276AG* had a lower risk of T2DM [*OR*(95%*CI*)= 0.75(0.57-0.99), ***Table 8***]. However, from the stratification analysis of the two SNPs in the *RXR*-*α* gene, there was no significant difference in the magnitude of the associations of T2DM with regard to age, gender or BMI (data not shown).

**Table 7:** Stratified analysis of rs3856806 genotype of the PPAR-*γ* gene and T2DM susceptibility

Stratified characteristics		Cases [*n*(%)]			Controls [*n*(%)]				CC	CT		TT	
		CC	CT	TT	CC		CT	TT		p*	*OR* (95%*CI*)*	P*	*OR* (95%*CI*)*
Sex	Male	316(59.0)	190(35.4)	30(5.6)	282(54.7)		194(37.6)	40(7.8)	1.00	0.180	0.84(0.64-1.09)	0.026	0.56(0.33-0.93)
	Female	350(61.5)	183(32.2)	36(6.3)	330(55.8)		220(37.2)	41(6.9)	1.00	0.076	0.79(0.61-1.03)	0.761	0.93(0.56-1.53)
Age(yr)	≤50	178(59.5)	106(35.5)	15(5.0)	139(49.6)		100(35.7)	41(14.6)	1.00	0.370	0.85(0.59-1.22)	0.000	0.25(0.13-0.50)
	>50	471(60.9)	253(32.7)	50(6.5)	472(57.2)		314(38.1)	39(4.7)	1.00	0.048	0.81(0.65-1.00)	0.269	1.29(0.84-2.01)
BMI	Normal	261(59.3)	152(34.5)	27(6.1)	312(55.7)		206(36.8)	42(7.5)	1.00	0.354	0.88(0.67-1.15)	0.538	0.85(0.51-1.43)
	Overweight	274(59.3)	159(34.4)	29(6.3)	230(56.2)		149(36.4)	30(7.3)	1.00	0.338	0.87(0.65-1.16)	0.424	0.80(0.46-1.37)
	Obesity	114(67.1)	49(28.8)	7(4.1)	59(51.8)		48(42.1)	7(6.1)	1.00	0.008	0.49(0.29-0.83)	0.180	0.47(0.15-1.42)

*adjusted for age, sex and body mass index (BMI).

**Table 8:** Stratified analysis of rs17817276 genotype of the *PPAR*-*γ* gene and T2DM susceptibility

Stratified characteristics		Cases [*n*(%)]			Controls [*n*(%)]				AA	AG		GG	
		AA	AG	GG	AA		AG	GG		p*	*OR* (95%*CI*)*	P*	*OR* (95%C/)*
Sex	male	393(73.6)	131(24.5)	10(1.9)	338(65.5)		155(30.0)	23(4.5)	1.00	0.045	0.75(0.57-0.99)	0.011	0.36(0.16-0.80)
	female	411(72.6)	144(25.4)	11(1.9)	437(74.1)		138(23.4)	15(2.5)	1.00	0.590	1.08(0.81-1.44)	0.393	0.70(0.30-1.60)
Age(yr)	≤50	209(69.9)	86(28.8)	4(1.3)	190(68.1)		82(29.4)	7(2.5)	1.00	0.645	0.91(0.62-1.34)	0.348	0.54(0.15-1.97)
	>50	571(74.3)	181(23.5)	17(2.2)	584(70.8)		210(25.5)	31(3.8)	1.00	0.341	0.89(0.71-1.13)	0.024	0.48(0.26-0.91)
BMI	normal	334(76.3)	97(22.1)	7(1.6)	394(70.5)		146(26.1)	19(3.4)	1.00	0.070	0.76(0.56-1.02)	0.052	0.41(0.17-1.01)
	overweight	325(70.8)	126(27.5)	8(1.7)	285(69.7)		112(27.4)	12(2.9)	1.00	0.956	0.99(0.73-1.34)	0.372	0.66(0.26-1.65)
	obesity	119(70.0)	47(27.6)	4(2.4)	82(71.9)		25(21.9)	7(6.1)	1.00	0.566	1.19(0.66-2.13)	0.184	0.42(0.12-1.51)

*adjusted for age, sex and body mass index (BMI).

## DISCUSSION

In this study, we selected four tagging SNPs in the *PPAR*-*γ* and *RXR*-*α* gene to investigate the association with the risk of T2DM in a Chinese population using a moderate sample size of 1,105 diabetic cases and 1,107 diabetes-free controls. In the single locus analysis, we observed statistically significant differences between case patients and control subjects in genotype distribution of *rs17817276GG*, *rs3856806CT*, and *rs3856806CT/TT* in the *PPAR*-*γ* gene (adjusted *P* = 0.010, 0.031 and 0.016, respectively). PPAR-γ is a member of the nuclear receptor family of ligand-activated transcription factors that heterodimerizes with RXR to regulate gene expression. The human gene encoding PPAR-γ has been localized to chromosome 3 (3p25). PPAR-γ, which is located primarily in the adipose tissue, lymphoid tissue, colon, liver and heart is thought to regulate adipocyte differentiation and glucose homeostasis. PPAR-γ has been implicated in the pathology of numerous diseases including obesity, diabetes, atherosclerosis and cancer. Up to now, there have been very few studies investigating the association between *rs3856806C/T* of the *PPAR*-*γ* gene with the risk of T2DM, and the conclusions are conflicting. In these studies, only one of the domestic research groups found a statistically signiﬁcant association of *rs3856806C/T* polymorphism with the obese and overweight T2DM patients[19]. It was reported that polymorphism C1431T of exon 6 of *PPAR*-*γ* (*rs3856806*) was associated with lower diabetes risks[14]. In a Singaporean population study, Tai *et al*.[20] found that Singaporeans carrying CC homozygote, polymorphism of rs3586806C>T had a reduced risk of diabetes. Doney *et al*.[21] found that the T allele of rs3586806 could reduce the risk of T2DM in Scotland, but Evans *et al*.[22] did not find a statistically signiﬁcant association between the rs3586806C>T polymorphism and T2DM in a German population study. We failed to find those results in our study. This may be due to differences in ethnicity and type 2 diabetes susceptibility variants. We present data that suggest an association between rs3856806C>T and T2DM in Han Chinese. The allele frequency for the C allele was 0.756 and 0.244 for T allele, demonstrating higher T-allele frequency than in previous studies[14],[23],[24] and similar frequency to that in other studies[25],[26]. There was a statistically significant difference in terms of rs3856806 and rs17817276 genotype frequencies between the two groups.

Furthermore, in the stratified analysis, we found that the reduced risk of T2DM associated with rs3856806C>T SNP in the *PPAR*-*γ* gene was more pronounced among the elderly (> 50 years old) and obese subjects carrying the CT genotype; similarly, we found that the lower risk of T2DM associated with rs3856806 TT genotype was more pronounced among males and younger subjects (≤50-year-old). Few studies[23],[24],[27]–[29] analyzed the effects of the C1431T polymorphism, and none of them found signiﬁcant associations of this gene variant with glucose or lipid-related variables. However, one investigation in Caucasian subjects reported lower concentrations of apolipoprotein B and reduced coronary artery disease risk in carriers of the T-allele compared with CC homozygotes[24]. Another study in Caucasians reported higher leptin levels in obese subjects bearing the T allele[23], but only one of these studies[29] found a statistically signiﬁcant association between the rs3586806C>T polymorphism and BMI, with subjects bearing the T allele having a higher mean BMI[30]. Furthermore, we found that a risk reduction associated with the *PPAR*-*γ* variant genotypes was more pronounced for obese patients, suggesting that patients with different BMI have different etiologies, not only in relation to environmental risk factors but also in genetic susceptibility. Because of the small sample size in obese subjects, their findings were preliminary and need to be validated in further studies with larger sample sizes in the obese population. In summary, our findings are not completely identical to those of other domestic and foreign research groups. This inconsistency may be due to the results of different genetic backgrounds of the subjects, sample sizes and different sample inclusion criteria or other factors.

We further evaluated the association of the rs17817276GG variant genotype with T2DM risk by selected variables. As shown in ***Table 8***, the effect of GG variant genotypes was more evident in male and older subjects. Compared with the rs17817276AA genotype, male subjects carrying the AG genotype had a significantly reduced risk. So far, studies investigating the association between rs17817276A/G of the *PPAR*-*γ* gene and the risk of T2DM have not been reported yet.

Interestingly, we found that haplotype GT was less frequent in cases than in controls (*P* < 0.001 for both two-sided χ^2^-test). Thus, the study of haplotypes suggested that the two SNPs (rs3856806 and rs17817276) may jointly reduce T2DM risk. Haplotype GT, with variant allele G of rs17817276A/G and T of rs3856806C/T, could decrease the risk for T2DM compared with the most common haplotype AC [adjusted *P* < 0.001, OR (95% CI) = 0.13(0.06-0.31)]. Therefore, rs17817276G and rs3856806T were responsible for the association. However, the exact mechanism of the haplotype effect is not fully understood. Therefore, potential locus-locus interactions between SNPs of *PPAR*-*γ* and *RXR*-*α* need to be further elucidated in future studies.

PPAR-γ acts as a nuclear receptor-transcription factor by forming a heterodimer with RXR. Coexpression of PPAR-γ and RXR-α increased human adiponectin promoter activity, although expression of PPAR-γ or RXR-α alone failed to enhance the promoter activity[12]. The current views have identified a functional PPAR-responsive element (*PPRE*) in human adiponectin promoter. PPAR-γ is a member of the nuclear receptor family of ligand-activated transcription factors that heterodimerize with the retinoid X receptor (*RXR*) to regulate gene expression[31]. In adipocytes, point mutation in the PPRE markedly reduced the basal transcriptional activity and completely blocked thiazolidinedione-induced trans-activation of adiponectin promoter[11]. As presented, patients with TT genotype of rs3856806 had a higher serum level of adiponectin than those with the CC and CT genotype (*P* = 0.031,0.038). Thus, we considered that rs3856806 of *PPAR*-*γ* was associated with serum adiponectin level.

Like all other case-control studies, inherent biases existed in this study. Because the cases were from hospitals, the study subjects may not be fully representative of the general population. So this limitation may influence the observed associations. Although less than 5% of each locus of the DNA samples failed genotyping, this may have caused some selection bias. The associations between *PPAR*-*γ*/*RXR*-*α* variants and T2DM risk were estimated by computing the ORs and 95% CIs from logistic regression analysis with adjustment for age, sex and BMI. Other limitations relate to the complex functions of the *PPAR*-*γ* gene and to the lack of confirmation of our findings in other populations.

In conclusion, HapMap-based tagging of SNPs and haplotypes in the *PPAR*-*γ* gene were associated with risk of T2DM. Our study provides evidence that *PPAR*-*γ* rs385680C>T and rs17817276 A>G polymorphisms may be a genetic susceptibility marker for T2DM in a Chinese Han population. Our findings need to be validated by further functional studies as well as well-designed larger molecular epidemiological studies with diverse ethnic populations.
